# Online Social and Professional Support for Smokers Trying to Quit: An Exploration of First Time Posts From 2562 Members

**DOI:** 10.2196/jmir.1340

**Published:** 2010-08-18

**Authors:** Peter Selby, Trevor van Mierlo, Sabrina C Voci, Danielle Parent, John A Cunningham

**Affiliations:** ^4^Evolution Health Systems IncTorontoCanada; ^3^Ontario Tobacco Research UnitTorontoCanada; ^2^University of TorontoTorontoCanada; ^1^Centre for Addiction and Mental HealthTorontoCanada

**Keywords:** Internet, social support, addiction, treatment, tobacco, WATI

## Abstract

**Background:**

Both intratreatment and extratreatment social support are associated with increased rates of smoking cessation. Internet-based social support groups have the capability of connecting widely dispersed groups of people trying to quit smoking, making social support available 24 hours a day, seven days a week, at minimal cost. However, to date there has been little research to guide development of this particular feature of Web-assisted tobacco interventions (WATIs).

**Objective:**

Our objectives were to compare the characteristics of smokers who post in an online smoking cessation support group with smokers who do not post, conduct a qualitative analysis of discussion board content, and determine the time it takes for new users to receive feedback from existing members or moderators.

**Methods:**

Data were collected from StopSmokingCenter.net version 5.0, a WATI equipped with an online social support network moderated by trained program health educators that was operational from November 6, 2004, to May 15, 2007. Demographic and smoking characteristics for both users and nonusers of the online social support network were analyzed, and qualitative analyses were conducted to explore themes in message content. Posting patterns and their frequency were also analyzed.

**Results:**

During the study period, 16,764 individuals registered; of these, 70% (11,723) reported being American. The mean age of registrants was 38.9 years and 65% (10,965) were female. The mean number of cigarettes smoked was 20.6 per day. The mean score for the 41% (6849) of users who completed the Fagerström Test for Nicotine Dependence was 5.6. Of all registered members, 15% (2562) made at least one post in the online social support network; 25% of first posts received a response from another member within 12 minutes, 50% within 29 minutes. The most frequent first posts were from recent quitters who were struggling with their quit attempts, and most responses were from members who had quit for a month or more. Differences in demographic and smoking characteristics between members who posted on the support group board at least once and those who did not post were statistically but not clinically significant.

**Conclusions:**

Peer responses to new users were rapid, indicating that online social support networks may be particularly beneficial to smokers requiring more immediate assistance with their cessation attempt. This function may be especially advantageous for relapse prevention. Accessing this kind of rapid in-person support from a professional would take an inordinate amount of time and money. Further research regarding the effectiveness of WATIs with online social support networks is required to better understand the contribution of this feature to cessation, for both active users (posters) and passive users (“lurkers”) alike.

## Introduction

Extensive evidence exists to prove the effectiveness of several traditional behavioral interventions for smoking cessation, including brief or intensive advice, individual or group counseling, tailored self-help, and telephone quitlines [1,2]. As access to the Internet continues to expand globally and an increasing number of individuals turn to the Internet to search for health information [3], a growing number of individuals are likely to seek help on the Internet with quitting smoking in place of or as an adjuvant to more traditional forms of treatment. The wide reach of these Internet-based interventions thus provides an opportunity to impact tobacco use at a population level [4]. A review of several randomized controlled trials concluded that tailored, interactive, Internet-based interventions significantly increase abstinence rates compared with untailored written or Web-based materials, and their effectiveness appears to be similar to intensive face-to-face counseling [5]. However, insufficient evidence and considerable heterogeneity in design at present prevent any reliable conclusions or recommendations regarding the effectiveness of Web-assisted tobacco interventions (WATIs) to be made [1,5]. Thus, further research is necessary to characterize the different features of WATIs and how they are experienced by users in order to determine for whom and by what mechanisms WATIs may be effective.

### Online Social Support for Health

Based on evidence from traditional behavioral interventions, current cessation guidelines recommend that all smoking cessation interventions incorporate an element of social support [1]. Meta-analytic findings indicate that providing either intratreatment or extratreatment social support significantly increases the odds of smoking abstinence at follow-up (OR = 1.3, 95% CI = 1.1-1.6 and OR = 1.5, 95% CI = 1.1-2.1, respectively) [1]. Access to the Internet affords the possibility to connect individuals worldwide 24 hours a day, seven days a week, at minimal cost, eliminating barriers to in-person group participation due to factors such as childcare, disability, and employment.

Cutrona and Suhr [6] developed a coding scheme that classifies social support behavior under two broad categories: “nurturant” and “action facilitating.” Nurturant types of social support help the person cope with the situation without necessarily solving the problem. Subcategories include “emotional support” (eg, “I know how you feel. The crankiness was the worst part for me too, but it does pass”), “esteem support” (eg, “don’t let that slip get you down, I know you can do it”), and “social network support” (eg, “I am glad you are part of this support group, and we are here to help you”). Action facilitating social support, on the other hand, is usually subcategorized into “informational support” that intends to help solve the problem causing stress (eg, “you can drink a glass of water when you have a craving”) or “tangible support” (eg, “I will send you a book on smoking cessation medications and the name of an excellent specialist”). Several content analyses of posts on online communities for health conditions such as irritable bowel syndrome [7], Huntington’s disease [8], and HIV/AIDS [9], have utilized Cutrona and Suhr’s [6] coding scheme and identified that all five subtypes of social support are evident in posts, with informational and emotional support most frequently offered [7,8,9]. However, Coursaris and Liu [9] noted that not all post content fit into this social support framework, and thematic analysis identified an additional three themes that were seen as facilitating social support: sharing personal experiences, expressing gratitude, and offering congratulations.

In the addictions field, there have been very few published studies that have examined the content of posts to online social support groups. In a study of AlcoholHelpCentre.net (AHC) [10], analyses of 474 posts made by registered problem drinkers revealed that the most common themes were providing encouragement and suggestions to other forum members and expressing gratitude for support received. Of the 155 registered members of AHC, 32% made at least one post on the forum, and those who posted did not differ significantly on any demographic characteristics compared with those who did not post.

To date there has only been one published qualitative analysis of posts to an online smoking cessation forum, part of the primarily French-language website, www.Stop-tabac.ch, a nonprofit WATI [11]. Burri [11] analyzed all 1033 messages posted in April 2005 by 97 ex-smokers who had quit smoking within the past 6 months. The most frequent theme identified among posts was providing emotional support and encouragement, followed by personal stories and opinions, congratulations to quitters, commonplace remarks often not related to tobacco, and expressing gratitude to other forum members. Discussing smoking cessation medications, giving practical advice and tips, and asking for information or emotional support were among the least common themes.

However, a number of features of the study by Burri and colleagues [11] limited its generalizability to other online support groups for smoking cessation. The forum was not moderated by a professional, and members were segregated according to their “stage of change” [12]. Therefore, the findings only reflected the experiences of recent quitters in the “action” stage, who comprised the sample. Segregation by stage of change may have prevented these members from observing or communicating with other members at different stages, and other online support boards that do not share this design may exhibit differences in interactions between members.

### Current Study

The purpose of the current study was to explore seeking and providing social support on a moderated online smoking cessation support group board. In particular, we sought to answer the following research questions: (1) Are there any differences in demographic or smoking-related characteristics that differentiate smokers who choose to post on an online smoking cessation discussion board and those who do not choose to post? (2) What topics prompt someone to make a first post on a smoking cessation discussion board, and do specific topics prompt members to post more quickly after joining an online community? (3) Are any topics more frequently or more quickly responded to by other members of an online support group? The current paper attempts to answer these questions using data collected from the English language StopSmokingCenter.net (SSC), version 5.0, a free-access WATI with both a self-help behavior change program and an online social network moderated by trained health educators.

## Methods

### Description of the Program

**Figure 1:**
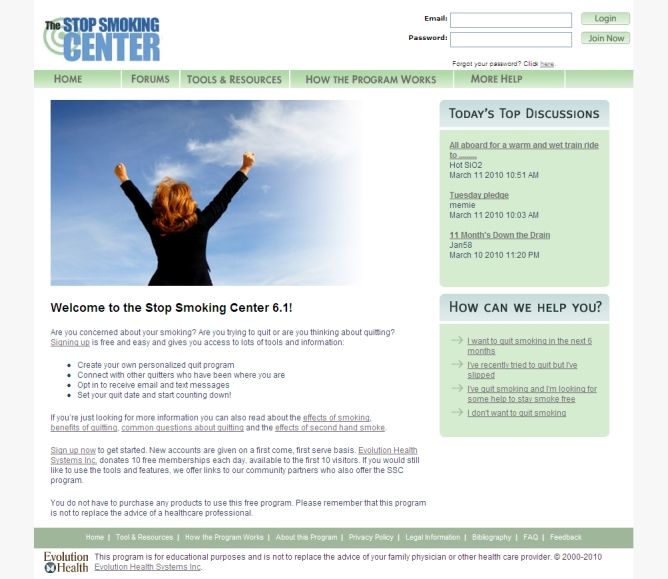
Screenshot of StopSmokingCenter.net 6.1 home page

Version 1.0 of the SSC was officially launched on September 28, 2000. Since launch, the program has been updated five times, with the most recent version of the SSC (6.1) released on January 1, 2008 (see Figure 1). At the time of data collection, the life cycle of version 5.0 had been longer than version 6.0 and 6.1 and thus provided a greater quantity of data for analysis; therefore, data for the current study were collected from the lifecycle of SSC version 5.0, which lasted from November 6, 2004, through May 15, 2007 (the study period). Version 6.1 differs from version 5.0 in the information architecture (IA) of the program's behavior change program. Version 5.0 was based on tunnel IA design, which guided users through a strict, step-by-step series of interactive exercises.Version 6.1 of SSC utilizes a free-form matrix IA design, which allows users to freely explore all program elements and self select interactive exercises, information, and tools. The decision to modify the program from the tunnel IA design to the free-form IA design was based on current literature outlining how individuals utilize eHealth programs [13] and results from usability testing at the Centre for Global eHealth Innovation in Toronto, Ontario. Recent evidence indicates that the free-form IA design increases usability [14]. However, it is important to note that version 5.0 utilized the same support group software as version 6.1 of the SSC, and users of version 5.0 were permitted to post in the support group at any time following program registration, as in version 6.1. Therefore, although there are differences in version 6.1 in access to behavior change exercises, information, and tools, access to the support group of the program was the same in version 5.0 as in version 6.1.

There were no fees to access the program; however, to participate and post messages in the program, registrants must have agreed to abide with a user agreement and consented to the use of their anonymous data for research purposes. The program does not sell, advertise, or promote any products, and user data were not sold to any third party. Registration enabled a unique ID number to be assigned to each member and allowed for tailored information to be provided to the member. Following registration, a unique password was sent to the member’s email address. The unique password permitted the member access to all of the program’s tools and services.

Following registration, members were able to use all of the tools within the tailored quit program, participate in the online social support group, email questions to the program’s trained moderators (health educators), receive inspirational emails, and chat with other members via the program’s Quitting Buddies Instant Messaging program. If a health care professional or a researcher registered with the program for review purposes, they were asked to endorse a second checkbox to indicate their health care professional or researcher status and their data were discarded from the database. Unregistered visitors had the ability to view and search all posts and all message threads within the program’s support group. However, to participate in discussions or use other features of the program, registration was required.

**Figure 2:**
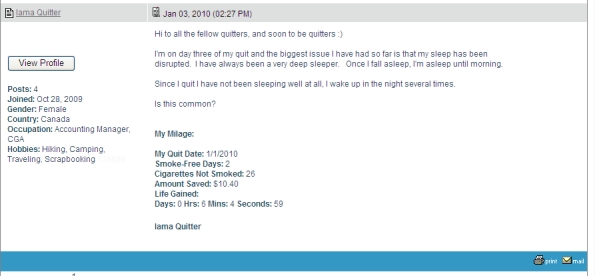
Screenshot of a sample StopSmokingCenter.net 6.1 support group post

Once members endorsed the user agreement and accessed their tailored program, they had the additional ability to upload specific information to their personal profile, which could be displayed as part of their support group post(s). The personal profile was optional, but if used, personal profile information was then in the public domain and could be viewed by other members as well as by users who had not registered, known as “lurkers.” Members could provide within their profile an avatar or uploaded image as well as personal information including their sex, age, country of origin, occupation, and hobbies. Members could also add a tagline or signature to their posts and could select to display all, some, or none of their personal profile. In addition to optional personal profile information, members’ usernames, dates they joined SSC (registered), and numbers of posts to date were automatically displayed in their support group posts (see Figure 2).

All posts made within the support group boards were published instantaneously but were reviewed by the program’s health educators via WebTriage. WebTriage enabled health educators to review, approve, edit, or delete posts (see Cunningham et al [10] for more details). Health educators could also communicate directly with members via email to ensure appropriate behavior and edit or delete member posts. All program health educators were paid employees of Evolution Health Systems Inc and were trained to only give brief, behavioral advice and were instructed to not provide one-on-one counseling, discuss specific instances of medication use, or provide psychiatric advice.

### Ethical Considerations

All members, having completed the registration process, consented to the use of their anonymous data for research purposes. During registration, an explanation was provided to participants as to how their information would be used and how privacy would be maintained. Registration or log-in was not required to access the support group boards, therefore all posts were in the public domain. To further protect member privacy, anonymity was promoted and potential members were encouraged at registration to use free email services such as Hotmail, Yahoo! Mail, or Gmail. The current study was approved by the Research Ethics Board at the Centre for Addiction and Mental Health (#456/2007).

### Measures and Data Collection

Data collected during the study period were extracted from the program’s customized structured query language (SQL) server database. Information on demographics (age, gender, and country of residence) and smoking behavior (number of years smoked and number of cigarettes smoked per day) collected at registration was extracted.

One of the exercises members had the option of completing following registration was the Fagerström Test for Nicotine Dependence (FTND) [15], a widely used self-report measure of nicotine dependence. The FTND is composed of six items including questions assessing number of cigarettes smoked per day, time to first cigarette in the morning, smoking when ill, and difficulties refraining from smoking where prohibited. High levels of nicotine dependence are classified by scores of 6 or higher. The coefficient alpha for the current sample was .63, similar to the value reported by the scale developers (alpha = .61) [15]. Scores were extracted for all members who opted to complete the FTND.

Information regarding quit status was not requested at the time of registration. However, the majority of posts on the support group boards contained information regarding quit status. In addition to content posted (eg, “I’ve been smoke-free for 3 days now”), the optional “My Quit Date” exercise allowed members to automatically append quit date information onto each post as part of their signature (eg, quit date, number of smoke-free days) (see Figure 2). Using both of these sources of information, all first posts were coded for the quit status of the member who posted. Similarly, the content of first replies to first posts was coded to determine the quit status of the member who posted the reply.

For each member, date and time values were extracted from the SQL server database for (1) completion of the registration process, (2) first post, where applicable, and (3) first reply to first post, where applicable. This allowed for calculation of time elapsed (in hours) between registration and first post and between first post and first reply. In addition, the content of all first posts and first replies was extracted for qualitative analysis.

### Data Analysis

The content of all first posts was analyzed using content analysis. Themes were identified using an inductive approach grounded in the data as opposed to a deductive approach guided by existing theory and/or predetermined categories or themes. The fourth author (DP), an employee of Evolution Health Systems Inc and a fourth year nursing student, coded the content of each post and identified a list of themes. Many posts contained more than a single theme.

Background information (ie, demographic and smoking variables) and themes for first posts were entered into a database for analysis. Chi-square and *t* tests were computed to compare groups on categorical and continuous variables, respectively. Time to first post and time to reply variable distributions were significantly skewed and kurtotic; therefore, nonparametric Mann-Whitney U tests and Kruskal-Wallis tests were conducted to determine whether time to post or reply varied by absence or presence of a theme or a member’s quit status, respectively. Medians and ranges are reported in place of means and standard deviations for nonnormally distributed variables. Statistical analyses were conducted with SPSS version 15.0 (SPSS Inc, Chicago, IL). In light of the large sample size and number of comparisons being made, significance was set at the more stringent level of *P* < .01 to reduce type I error.

## Results

### Demographic and Smoking Characteristics

During the study period 16,764 smokers (“members”) had registered with SSC version 5.0. Of these 16,764 registrants, 15.3% (2562) made at least one post to the support group boards (“posters”), while 84.7% (14,202) did not post (“nonposters”). (See Table 1 for demographic characteristics of posters and nonposters.) The average smoker who registered with SSC was 39 years old, had smoked for approximately 20 years, smoked a pack a day, and had a moderate to high level of nicotine dependence. The majority of smokers were female. Although, there were no clinically significant differences between posters and nonposters, the completion rate of the FTND was almost double among posters compared with nonposters. This likely reflected greater engagement in the process of smoking cessation by those who posted.

**Table 1:** Demographic and smoking characteristics of registered members of StopSmokingCenter.net version 5.0 (November 6, 2004, through May 15, 2007)

		All Registered Members	Posted On Support Group Boards	Did Not Post On Support Group Boards	*P* Value^a^
**Demographic Characteristics**		(n = 16,764)	(n = 2562)	(n = 14,202)	
	Female, % (n)	65.4 (10,965)	70.1 (1795)	64.6 (9170)	< .001
	Age (years), mean (SD)	38.9 (11.3)	40.4 (10.8)	38.7 (11.3)	< .001
**Country of residence**					< .001
	United States, % (n)	69.9 (11,723)	73.5 (1882)	69.3 (9841)	
	Canada, % (n)	12.6 (2104)	12.3 (315)	12.6 (1789)	
	United Kingdom, % (n)	7.1 (1191)	6.2 (159)	7.3 (1032)	
	Other, % (n)	10.4 (1746)	8.0 (206)	10.8 (1540)	
**Smoking characteristics**					
	Years smoked, mean (SD)	19.9 (11.2)	21.6 (11.0)	19.5 (11.2)	< .001
	Cigarettes per day, mean (SD)	20.6 (10.6)	22.2 (10.9)	20.4 (10.5)	< .001
	Completed FTND, % (n)	41.0 (6849)	66.7 (1708)	36.2 (5141)	< .001
	FTND score^b^, mean (SD)	5.6 (2.3)	5.8 (2.2)	5.5 (2.3)	< .001
	High level of nicotine dependence (FTND score > 6)^b^, % (n)	54.9 (3759)	58.3 (996)	53.7 (2763)	< .001

^a^ Differences in demographic and smoking characteristics are statistically significant, but are not clinically significant.

^b^ Based on subsample that completed the FTND (n = 6849)

### First Posts to Online Support Group

There were 2562 first posts to the online support group. A complete list of the most relevant first post coding themes—overall and according to quit status—is presented within Table 2. The most common theme that emerged overall was seeking support or advice with quitting.

**Table 2:** Themes in first posts to support group by members of StopSmokingCenter.net version 5.0 (November 6, 2004, through May 15, 2007)

		Quit Status			
	Total^a^ (n = 2562)	Not Quit (n = 637)	Quit ≤ 1 month (n = 1401)	Quit > 1 month (n = 228)	*P* Value^b^
Theme of First Post	% (n)	% (n)	% (n)	% (n)	
Struggling with quitting and seeking support or advice	28.0 (717)	17.4 (111)	34.6 (485)	32.9 (75)	< .001
Responding to another member’s post or comment	17.6 (451)	7.4 (47)	16.8 (236)	32.5 (47)	< .001
Question or comment about cravings or triggers	15.3 (391)	10.2 (65)	19.5 (273)	11.0 (25)	< .001
Question or comment about withdrawal symptoms or postcessation weight gain	16.1 (413)	5.7 (36)	20.7 (290)	23.2 (53)	< .001
Discussing need to quit for own health or the health of others	13.6 (348)	22.1 (141)	12.8 (179)	6.1 (14)	< .001
Sharing a tip or strategy about quitting	6.8 (174)	0.5 (3)	6.5 (91)	20.2 (46)	< .001
Had a slip or relapse	6.2 (158)	5.8 (37)	6.7 (94)	4.8 (11)	.47
Question or comment about nicotine replacement therapy or other quit aid	4.5 (115)	5.2 (33)	3.5 (49)	6.6 (15)	.04
Encouraged to quit by family member or friends	3.7 (95)	5.0 (32)	3.6 (51)	1.8 (4)	.07
Joining in a “stats parade” or “rally cry” to celebrate achievement (eg, number of days smoke free)	1.7 (44)	0 (0)	1.8 (25)	5.3 (12)	< .001
Expressing a desire to quit	1.2 (32)	2.2 (14)	1.3 (18)	0 (0)	.04
Financial motivation to quit	1.1 (29)	2.0 (13)	1.1 (16)	0 (0)	.048
Having technical difficulty with the website	0.7 (19)	0.3 (2)	0.6 (8)	0.4 (1)	.74
Surprised or concerned about how easy the first few days had been	0.2 (6)	0 (0)	0.4 (6)	0 (0)	.16
Told to quit by health care professional	0.2 (6)	0.3 (2)	0.2 (3)	0 (0)	.68

^a^ Total includes members whose quit status could not be determined.

^b^ Based on subsample with quit status information available (n = 2266)

Approximately 54.7% (1401/2562) of members who posted a message on the support group boards had recently quit smoking (ie, ≤ 1 month prior), 8.9% (228/2562) had quit more than 1 month previously, and 24.9.% (637/2562) had not quit smoking yet but had set a future quit date or expressed a desire to quit. Quit status could not be ascertained for 11.6% (296/2562) of support group posters.

**Figure 3:**
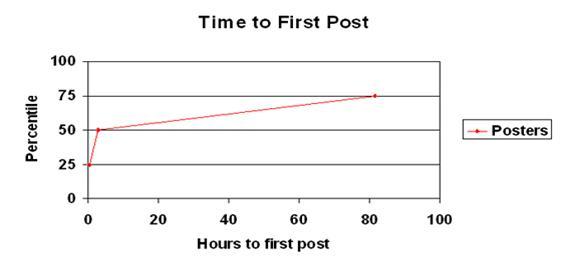
Time to first post on support group by members of StopSmokingCenter.net version 5.0 (November 6, 2004, through May 15, 2007)

Considering the necessary steps to register, navigate to the support group, and write a personal message, it is interesting to note that 25% of all first posts to the support group occurred within 20 minutes after registration, 50% of first posts occurred within 3 hours of registration, and 75% of first posts occurred within 81 hours of registration (see Figure 3).

Presence (versus absence) of the theme discussing the need to quit for one’s own health or the health of others was associated with a faster time to post (median 1.0 hours, range 0 - 13,030 vs median 4.7 hours, range 0-17,524; Mann-Whitney U = 300,598; *P* < .001). In contrast, presence of several themes associated with providing support to others were associated with a slower time to first post, namely: responding to another member’s post or comment (median 29.3 hours, range 0-13,827 vs median 1.7 hours, range 0-17,524; U = 323,156, *P* < .001); sharing a tip or strategy about quitting (median 72.0 hours, range 0-15,638 vs median 2.3 hours, range 0-17,524; U = 123,133, *P* < .001); and joining in a “stats parade” or “rally cry” (median 71.3 hours, range 0-11,161 vs median 2.8 hours, range 0-17,524; U = 35,080, *P* < .001) whereby members join in celebration and congratulations of another member’s achievements (eg, number of days smoke-free). Stats parades and rally cries could have occurred regularly (eg, every Friday) or spontaneously.

Time to first post varied significantly by quit status (Kruskal-Wallis H_2_= 118.2, *P* < .001). Follow-up post-hoc Mann-Whitney U tests revealed that members that had not quit (median 0.9 hours, range 0-8,448) posted sooner than members who had quit for less than one month (median 10.0 hours, range 0-17,089; U = 348,520, *P* < .001), or more than one month (median 178.6 hours; range 0-17,518; U = 41,143, *P* < .001). Members who had quit less than one month previously were also faster to post compared with members who had quit for more than thirty days (U = 111,611, *P* < .001).

### First Replies

A total of 79.5% (2036/2562) of replies to first posts were from other members. An additional 15.6% (399/2562) of replies were from health educators. Only 2.7% (68/2562) of posts did not receive a response. The remaining 2.3% (59/2562) of “replies” were made by the same member who originally posted (eg, adding more information or “bumping” their post) and were excluded from further analyses.

Almost half (48.5% or 33/68) of all posts that did not receive a reply from either another member or moderator had been posted in response to another member’s thread. The second most prevalent theme among posts that did not receive a reply was sharing a tip or strategy about quitting (23.5% or 16/68). Both of these themes were significantly more prevalent among posts that did not receive a reply than among posts that did receive a reply at the *P* < .001 level (χ^2^_1_ = 46.62 and χ^2^_1_ = 30.31, respectively). No other first post themes were significantly associated with a greater or lower likelihood of receiving a reply or not. Examining responses from members only (ie, excluding moderator responses), no theme was associated with a lower likelihood of receiving a response when it was present versus absent within a first post. However, first posts that were joining in a “stats parade” or “rally cry” were significantly more likely to receive a reply from another member (2.3% vs 19.0%; χ^2^_1_= 7.92, *P* = .005) compared with posts that did not contain this theme.

**Figure 4:**
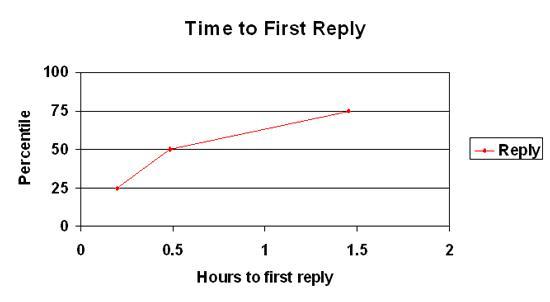
Time to first reply to first post from a support group member of StopSmokingCenter.net version 5.0 (November 6, 2004, through May 15, 2007)

Replies from other support group members were quick, with 25% of first posts receiving a reply within 12 minutes, 50% within 29 minutes, and 75% within 1 hour and 30 minutes (see Figure 4). Time to reply to first posts from moderators was very similar in length, with 25% received within 14 minutes, 50% within 33 minutes, and 75% within 1 hour and 27 minutes. 

Struggling and seeking support or advice was the only theme associated with a faster time to reply from another support group member (median 0.4 hours, range 0-8622 vs median 0.5 hours, range 0-1,386; U = 378,756, *P* <.001). Themes associated with a slower time to reply by other support group members when they were present (versus absent) within a first post were responding to another member’s post or comment (median 0.9 hours, range 0-1386 vs median 0.4 hours, range 0-8,622; U = 245,691, *P* < .001) and sharing a tip or strategy about quitting (median 0.8 hours, range 0-567 vs median 0.5 hours, range 0-8622; U = 105,534, *P* < .001) .

The majority of replies were made by members who had quit, 35.0% (713/2036) by members who had quit within the past month, 49.0% (997/2036) by members who had quit for more than 1 month but less than 1 year, and 6.6% (135/2036) by members who had quit for more than one year. Only 1.4% (28/2036) of members who posted a first reply had not quit. Quit status could not be determined for 8.0% (163/2036) of response posts. Quit status of members who posted a reply was not related to the themes of the first posts replied to. The quit status of members who replied was also not associated with the length of time to post a reply (H_3_= 3.86, *P* = .28).

## Discussion

Findings from the current study revealed that 15% of members of an online smoking cessation program chose to make a post on the support group boards. First posts were made relatively quickly—50% within three hours—and members most frequently conveyed that they were seeking support and advice. Provision of support was prompt, with 50% receiving a reply within 29 minutes. Responses were even faster when posts communicated that a member was seeking support, revealing that the support group board did function to provide members with an immediate source of support not available with most traditional interventions. While first posts were most often made by recent quitters or those planning to quit, those who had quit for a month or longer were more likely to reply and provide support to other members.

### Principal Results

Only a small minority of StopSmokingCenter.net members chose to actively participate on the support group boards by posting at least one message, suggesting that other self-help quit program components may have been more appealing. However, an additional unknown number of members may have benefited sufficiently from lurking and reading posts alone. More than half of lurkers on online discussion board communities report that “just reading/browsing is enough” as a reason for not posting [16]. Research has shown that lurkers gain many of the same benefits of online support groups as those who actively post, including developing a strong sense of community [17]. For example, a recent study of Dutch online support groups for breast cancer, fibromyalgia, and arthritis found no difference between lurkers and posters across a range of self-reported empowering outcomes such as being better informed, increased optimism and control, enhanced self-esteem, increased acceptance of the disease, and feeling more confident about treatment [18]. The only exception was that posters reported a greater enhancement of their social well-being compared with lurkers. Together these findings suggest that more individuals may utilize and benefit from online discussion boards than can be judged by examining the frequency of posts and number of individuals posting. The absence of substantive differences between posters and nonposters further supports the possibility that existing content on discussion boards could potentially have also met the needs of those who did not post.

In contrast, An and colleagues [19] found that active (ie, posting) but not passive (ie, reading) online community engagement was associated with increased smoking abstinence rates among WATI users at 6-month follow-up. Path analysis revealed that the association between active online community engagement and abstinence was accounted for in large part by increased use of interactive quitting tools and one-to-one messaging. Whether these findings indicated that active online community engagement promoted engagement with other features of the Web-based program, or vice versa, could not be determined. Nevertheless, these findings suggested that posting was associated with overall engagement with the Web-based program, which other research has established as a predictor of subsequent smoking cessation outcome [20]. Clearly, additional research is necessary to examine and compare the experiences of lurkers and posters on support boards, including the impact on smoking cessation outcomes.

Similar to findings from Stop-tabach.ch [11], more than half of the posters on the support group boards were recent quitters, and another quarter were in the preparation stages to quit. This suggests that people may require more support during the early stages of their quit process then during other times in their quit process. Because the quit status of smokers who did not post could not be determined in both studies, it is not clear whether or not nonposters or lurkers are at the same stage in the quit process as posters or if smokers are more likely to lurk at one stage and post at another. Smokers who were planning to quit were quicker to make a first post after registration, and discussing the need to quit for health reasons was also associated with a faster time to first post, suggesting a more immediate need for support for those who desire or are planning to quit.

Seeking support and advice was the most common theme identified in first posts among both recent and longer term quitters. Although less prevalent than among those who had quit, seeking support and advice was the second most common theme among members planning to quit. Thus, it is evident that one of the most common reasons that prompted a member to make a first post on the support group board was to seek help.

In addition to seeking support, several themes among first posts also revealed that provision of support to other members also prompted members to make a first post. Providing support was more evident among those who had already quit, particularly members who had quit for more than one month. Consistent with this was the finding that almost half of all replies to first posts were made by members who had quit for more than one month but less than one year, while only 1% of those who posted the first reply to a first post had not yet quit. Thus, it appears that members who had more experience with the quit process may have been more comfortable or inclined to provide advice or support to other members, providing posters a response from someone they could identify with while allowing the responder to model their behavior and reinforce their own commitment to quitting by articulating a response. Moreover, these posts and exchanges also allowed for vicarious learning by all viewers of the posts. Taken together, these findings reflect principles of social cognitive theory models in action [21]. Whether members who had quit for longer were more inclined to provide a particular type of support (eg, informational or emotional) was not examined in the current study but may have differed.

Despite the fact that those providing support were slower to make their first post, replies from other support group members were quite rapid. This demonstrates the almost immediate support that smokers can receive online, a significant advantage over several other types of more traditional supports. This feature may be particularly relevant for relapse prevention. However, particular features of first posts—date of registration, number of posts to date, and introductions (eg, “I’m new here”)—may trigger a more prompt response and may not be reflective of the timing of responses for all posts.

Replies to first posts were primarily from other support group members rather than health educators, and only 2.7% of posts did not receive a response. Almost three-quarters of posts that did not receive a reply from another member or moderator had been posted in response to another member’s thread or were sharing a tip or strategy about quitting. These types of messages may have been less likely to have been seeking a response, especially given that not all first posts began a new discussion thread. In fact, first posts that conveyed that the member was struggling and seeking support or advice received a significantly faster reply, while posts that were responding to another member’s post or comment or sharing a tip or strategy about quitting received slower replies. This suggests that other members responded to the needs of those who were seeking support and they received it faster.

### Limitations

One limitation of the current study was that the content of first posts was analyzed by one coder and resources were not available to determine interrater reliability with a second coder. Future research would benefit from including a second coder to enhance and verify the reliability of findings.

It is important to keep in mind when interpreting results of the current study that they reflect the content and timing of first posts and first replies and may not be generalizable to later (second, third, etc) posts or replies. Members that are more active on the discussion boards and go on to post second, third, or more posts may differ from those who are less active in posting on discussion boards. Furthermore, as smokers become more active on discussion boards, their posts may vary over time on several factors, including content and timing.

The support group analyzed in this paper has been operating for almost a decade, and the tone and style of messaging that has evolved in this particular support group, as well as the design of the broader behavioral program it is embedded within, may not be representative of other support groups, eHealth interventions, or WATIs. As well, the population who used the program were self-selected and found the program through their independent search initiatives and may not be representative of all smokers who have used WATIs or online support groups.

### Conclusions

When considering the most common themes of first posts, as well as the relatively short time to first post, the SSC support group may be regarded as a peer-to-peer social support tool for those who are struggling with quitting, particularly recent quitters, who require immediate support. Responses to first posts were timely and would have otherwise required the smoker to make an appointment to see a professional or track down a quit buddy. This provides smokers an opportunity to seek timely help which, if effective, may avoid a relapse back to smoking. Based on these findings, WATI developers and researchers may be inclined to create content and tools such as relapse prevention support and resources that appeal to this specific population. Further, as the program analyzed in this study was not advertised and the population was self-selected, there may be a large number of recent quitters who are seeking these types of services and would benefit from health promotion efforts that alert them to the availability of these types of programs.

## Abbreviations

FTND: Fagerström Test for Nicotine Dependence
IA: information architecture
SSC: StopSmokingCenter.net
SQL: structured query language
WATI: Web-assisted tobacco intervention
